# Physical Disability, Anxiety and Depression in People with MS: An Internet-Based Survey via the UK MS Register

**DOI:** 10.1371/journal.pone.0104604

**Published:** 2014-08-25

**Authors:** Kerina H. Jones, Philip A. Jones, Rodden M. Middleton, David V. Ford, Katie Tuite-Dalton, Hazel Lockhart-Jones, Jeffrey Peng, Ronan A. Lyons, Ann John, J. Gareth Noble

**Affiliations:** College of Medicine, Swansea University, Swansea, Wales, United Kingdom; Innsbruck Medical University, Austria

## Abstract

**Introduction:**

People with Multiple Sclerosis are known to have a relatively high prevalence of both anxiety and depression. Studies of the relationship between physical disability and mental health in people with MS have reported mixed results, showing the need for further work.

**Methods:**

Between May 2011 and April 2012, 4516 people completed the MSIS-29 (v.1) and HADS scales via the dedicated internet site of the UK MS Register within a 7 day time window. These responses were linked with basic demographic and descriptive data and analysed in SPSS (v.20).

**Results:**

The proportions of people experiencing anxiety or depression increased with physical disability such that 38.0% of respondents with low, and 66.7% with high disability reported at least mild anxiety, and 17.1% of people with low, and 71.7% with high disability experienced at least mild depression. The multiple regression model explained 18.4% of the variance in anxiety with MSIS-29-PHYS score being the strongest predictor of anxiety. The model for depression explained 37.8% of the variance with MSIS-29-PHYS score being the strongest predictor. Some of the other variables included showed negative associations with anxiety and depression, indicating that the influence of physical disability on mental wellbeing could be underestimated.

**Conclusions:**

This study indicates that there is a positive relationship between physical disability and anxiety and depression, that physical disability impacts on anxiety and depression to differing extents, and that the effects vary with gender, age, disease course and disease duration. We have shown that physical disability is a predictor of anxiety and depression, and that other factors may mask the extent of this effect. Whether the causes of anxiety and depression are reactive, organic or a combination, it is essential that mental wellbeing is given due attention in caring for people with MS so that all their health needs can be met.

## Introduction

Multiple Sclerosis is a chronic, inflammatory auto-immune condition resulting in unpredictable physical and psychological difficulties over the disease course. People with MS suffer higher rates of anxiety and depression than the general population [1]–[4] and they have been shown to have among the lowest quality of life scores across a range of chronic conditions [5]. Studies have examined mental well-being in chronic illnesses and MS is no exception [1], [2], [6], [7]. But, there have been mixed findings in studies of physical disability and mental health among people with MS, with some authors reporting a relationship, and some not finding one [8]–[13].

Advances are being made in therapeutic and biomedical MS research; however, there is an acknowledged lack of high quality evidence-based information about living with MS to inform research, policy and practice, and this led to the creation of the UK MS Register [14], [15]. The development of the UK MS Register was informed by a programme of qualitative research [16]–[19] and it has been designed on an innovative data model that brings together datasets from: NHS neurology clinics as people attend their out-patients appointments; sources of routinely-collected data, such as GP and hospital records; and people with MS directly via a dedicated internet portal by enrolling and providing responses to validated patient reported outcome measures (PROMs). The Register is built on the proven technologies and robust Information Governance arrangements established by the Secure Anonymised Information Linkage (SAIL) system, so that the data in the Register can be anonymously linked at the individual level and accessed for research whilst retaining privacy [20]–[22]. Data acquisition from clinics and routine sources is underway, subject to consent and data availability. The web portal was launched in May 2011 and, by April 2012, 8736 people with MS had enrolled, with further data collection underway as a continual process.

The portal hosts general health and disease assessment tools such as the Hospital Anxiety and Depression Scale (HADS) [23] and the EQ-5D [24], and disease-specific scales such as the MS Disease Impact Scale-29 (MSIS-29) [25]. These cover a range of topics such as: MS and mental well-being; impact of MS on daily life; lifestyle and health outcomes. Baseline information including: age, gender, date of diagnosis of MS and disease course is collected as part of the registration process and a questionnaire entitled ‘You, your MS and lifestyle’. The use of internet technologies in research is increasing, and we have shown that it is feasible to collect information in this way and to use it to characterize a cohort of people with MS [26]. We have also used this method to study the mental well-being of people with MS via the HADS [27], to show that the MSIS-29 can be administered via the internet and used to predict the likely impact of disability on taking an active part in the workforce [28], and how people with MS rate their own quality of life via the EQ5D [29].

The relationship between the physical and psychological impacts of MS measured via the MSIS-29 has been documented [25], [30], [31] and we have confirmed this in our cohort [28]. However, the MSIS-29 is not designed to distinguish between anxiety and depression and these conditions require specific courses of treatment and care. The MSIS-29 (v.1) consists of 29 questions divided into two components: 20 in the MSIS-29-PHYS for assessing the physical impact, and 9 in the MSIS-29-PSYCH for assessing the psychological impact, of MS. The responses are scored on a Likert scale (1–5, version 1) and summed to give a maximum of 100 on MSIS-29-PHYS and 45 on MSIS-29-PSYCH. These raw scores can be transformed to create two scales each spanning 0 to 100 and expressed as percentages [25], [32]. The HADS is a widely used measure of anxiety and depression, each of which are measured on a robust sub-scale of seven questions, scored from 0 to 3, giving possible maximum values of 21 [23]. The HADS has been validated for use with people who have MS [33]. Thus, by using the responses to the HADS and MSIS-29 scales, linkable within the MS Register, we are able to examine the relationships between the physical impact of MS and anxiety and depression. This is the only known study to use these scales to do this for a large cohort of people with MS. It is also novel because it is the largest known study of physical impact, anxiety and depression for MS, or any condition, conducted on data collected via the internet.

### Hypothesis and research question

There are mixed results of the relationships between physical disability and mental health in people with MS, and this needs further investigation [8]–[13]. Previous studies involving the general population and people with MS, have shown that anxiety and depression differ with various factors [27], [34]. Furthermore we showed that age and mental wellbeing can show a negative relationship, whereby there is a slight decrease in anxiety level with maturing age [27], [28]. Because of this, it is postulated that when physical disability is assessed in conjunction with mental wellbeing, there may differences in how its impact relates to anxiety and depression. Therefore, we set out to use the linked responses to the HADS and MSIS-29 scales to examine the relationships between physical disability, anxiety and depression. Bearing in mind likely modifying factors, we included analysis by gender, age, disease course and disease duration, to determine if, and the extent to which, physical disability impacts upon anxiety and depression.

## Methods

### Research ethics and governance

The UK MS Register received peer-review via MS Society mechanisms and it ethical approval from the South West – Central Bristol Research Ethics Committee (11/SW/0160) as a research database [35]. Under the conditions of this ethical approval, the datasets collected via the portal, the neurology clinics and routine sources can be anonymously linked at the individual level via the technologies and methodologies used by SAIL, provided that agreement to the portal terms of service (via the portal) and written informed participant consent (at the clinics) have been obtained. Although identifiable information is collected, this is only used to create data linkages. The functioning UK MS Register contains only anonymous data, but facilities are in place to re-contact participants to take part in further research [36]. The Register is open to research collaborations, subject to regulatory and governance requirements. The standard model is that agreed data sub-sets are accessed and analysed within a Safe Haven environment, with scrutiny of research outputs before release [20].

### Data collection and analysis

People with MS aged 18 years and over and living in the UK have been able to enrol on the web portal of the UK MS Register since its launch in May 2011. This study used the data collected up to April 2012, by which time 8736 people had joined. As we set out to compare responses to the HADS and MSIS-29, the 4516 responses to these scales completed within 7 days of each other were selected. These were collated with basic demographic and descriptive MS data and the resulting dataset was analysed in SPSS (v.20). As well as being used as continuous data, the HADS anxiety and depression components (with a maximum score of 21 for each) were classified into the accepted categories of normal (< = 7), mild (8–11), moderate (12–15) and severe (>15), using ≥8 as the threshold for indicating likely caseness [37], [38]. Within the MSIS-29 scale, raw scores (physical: 20 to 100, psychological: 9 to 45) and transformed scores (%) were both used for ease of reference, and the physical (MSIS-29-PHYS) and psychological (MSIS-29-PSYCH) scores were treated as separate sub-scales in all the analyses in accordance with the suggested guidance of Hobart and colleagues [25], [32] and our previous findings [28]. The physical scale was also divided into tertiles with thresholds of 33.3%, 66.7% and 100% to represent low, moderate and high disability scores, respectively. Descriptive statistics were used to characterize the cohort of people with MS. Frequencies were studied using categorised data, and chi-squared tests were used to assess independence. The Kruskal-Wallis one-way analysis of variance was used because the anxiety and depression scores failed the homogeneity of variances test for an ANOVA (Levene's statistic 20.503, df 4513, *p*<0.001; 41.910, df 4513, *p*<0.001). We had previously shown that physical and mental wellbeing can vary with certain factors such as gender, age, disease course and disease duration (time since confirmation of MS by a Neurologist) [27], [28], and so these variables were included in multiple regression models.

## Results

### Cohort description

The 4516 respondents were comprised of 28.9% men and 71.1% women, which is a ratio of 1 man: 2.46 women. The distribution of disease courses were: 14.8% primary progressive MS (PPMS), 62.1% relapsing-remitting MS (RRMS), 8.1% secondary progressive MS (SPMS) and 14.9% did not know their type of MS (DKMS) (N = 4422). The mean age of the respondents was 50.7 years (SE 0.17, SD 11.2) with a median of 51 years (IQR 16). The mean disease duration was 10.9 years (SE 0.15, SD 8.9) with a median of 9 years (IQR 12). The basic characteristics of this cohort have been described in more detail in a previous article [29]. The mean MSIS-29-PHYS score was 60.5 (50.6%) with a median of 62 (IQR 35) and the mean MSIS-29-PSYCH score was 24.8 (43.8%) with a median of 24 (IQR 14). The mean anxiety score was 8.03 with a median of 8, and the mean depression score was 7.37 with a median of 7. Fuller details of the MSIS-29 and HADS scores, including by gender and disease course, are shown in Table 1.

**Table 1:** The MSIS-29 and HADS scores of the cohort.

Categories	Mean	SD	SE	Median	IQR	Range
**MSIS-29-PHYS score (raw):**						
All	60.5	21.6	0.32	62.0	35	20 to 100
Men	63.5	21.0	0.58	66.0	33	20 to 100
Women	59.2	21.8	0.39	61.0	36	20 to 100
PPMS	70.0	17.9	0.70	72.0	27	22 to 100
RRMS	56.7	21.9	0.42	57.0	37	20 to 100
SPMS	73.6	16.0	0.84	74.0	22	28 to 100
DKMS	59.3	21.2	0.82	61.5	36	20 to 100
**MSIS-29-PHYS score (%):**						
All	50.6	27.0	0.40	52.5	44	0 to 100
Men						
Women						
PPMS	62.5	22.4	0.87	65.0	34	3 to 100
RRMS	45.9	27.3	0.52	46.3	46	0 to 100
SPMS	67.0	20.0	1.05	67.5	28	10 to 100
DKMS	49.1	26.5	1.02	51.9	45	0 to 100
**MSIS-29-PSYCH score (raw):**						
All	24.8	8.9	0.13	24.0	14	9 to 45
Men	24.9	8.9	0.25	24.0	14	9 to 45
Women	24.7	8.9	0.16	24	13	9 to 45
PPMS	24.7	8.6	0.33	24.0	13	9 to 45
RRMS	24.7	9.0	0.17	24.0	14	9 to 45
SPMS	26.1	8.7	0.46	25.0	13	9 to 45
DKMS	24.4	9.3	0.36	24.0	14	9 to 45
**MSIS-29-PSYCH score (%):**						
All	43.8	24.8	0.37	41.7	39	0 to 100
Men	44.3	24.8	0.69	41.7	39	0 to 100
Women	43.6	24.8	0.44	41.7	36	0 to 100
PPMS	43.5	23.9	0.93	41.7	36	0 to 100
RRMS	43.5	24.9	0.47	41.7	39	0 to 100
SPMS	47.5	24.1	1.27	44.4	36	0 to 100
DKMS	42.9	25.7	1.00	41.7	39	0 to 100
**HADS Anxiety score:**						
All	8.03	4.2	0.06	8.0	6	0 to 21
Men	7.51	4.3	0.12	7.0	7	0 to 21
Women	8.25	4.2	0.07	8.0	6	0 to 21
PPMS	7.41	4.2	0.17	7.0	6	0 to 20
RRMS	8.19	4.2	0.08	8.0	6	0 to 21
SPMS	8.08	4.3	0.23	8.0	6	0 to 21
DKMS	8.00	4.5	0.17	8.0	6	0 to 21
**HADS Depression score:**						
All	7.37	4.2	0.63	7.0	6	0 to 20
Men	7.90	4.3	0.12	8.0	6	0 to 20
Women	7.15	4.2	0.07	7.0	6	0 to 20
PPMS	8.00	4.2	0.16	7.0	6	0 to 20
RRMS	7.01	4.2	0.08	7.0	6	0 to 20
SPMS	8.64	4.1	0.22	8.0	7	1 to 20
DKMS	7.55	4.4	0.17	7.0	7	0 to 20

The physical (MSIS-29-PHYS) and psychological (MSIS-29-PSYCH) impact scores, assessed by the MSIS-29, and the anxiety and depression scores from the HADS of the cohort are shown. Values are included for all respondents, and by gender and disease course. N(all)  = 4516, N(men)  = 1302, N(women)  = 3198, N(PPMS)  = 654, N(RRMS)  = 2747, N(SPMS)  = 360, N(DKMS)  = 661). Small differences in totals for gender and disease course, compared to the overall total are due to some respondents (<2%) not providing these details.

### Proportions and levels of anxiety and depression by physical disability

**Figure 1:**
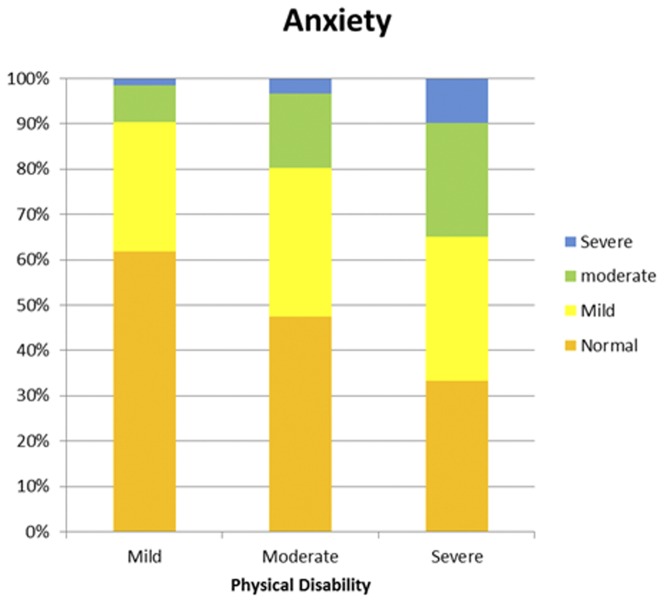
The proportions of people with low, moderate or high physical disability experiencing anxiety. The proportions of people reporting normal (< = 7), mild (8–11), moderate (12–15) or severe (>15) anxiety were assessed against low, moderate and high tertiles of physical disability are shown. It can be seen that greater numbers of the respondents reported symptoms of anxiety with increasing levels of disability. The numbers of people with higher levels of anxiety also increased.

**Figure 2:**
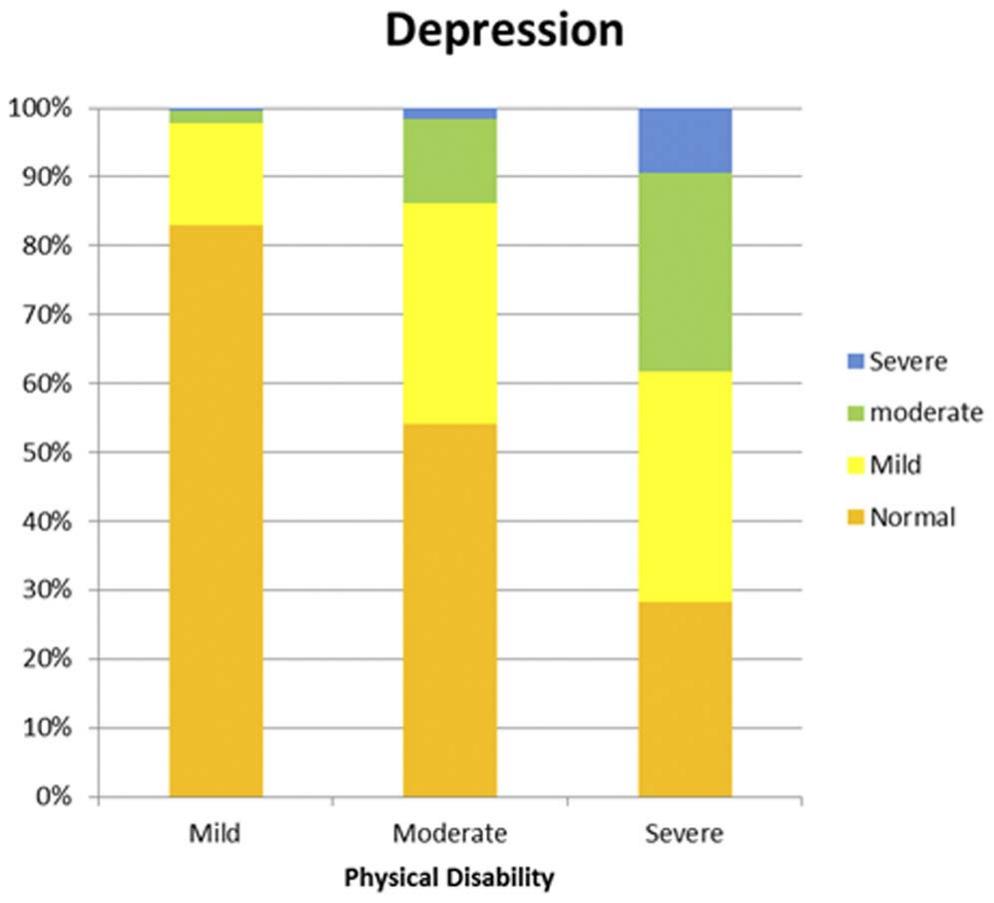
The proportions of people with low, moderate or high physical disability experiencing depression. This shows the proportions of people reporting normal (< = 7), mild (8–11), moderate (12–15) or severe (>15) depression were assessed against low, moderate and high tertiles of physical disability. Greater numbers of the respondents reported symptoms of depression with increasing levels of disability, and the numbers of people with higher levels of depression also increased.

**Figure 3:**
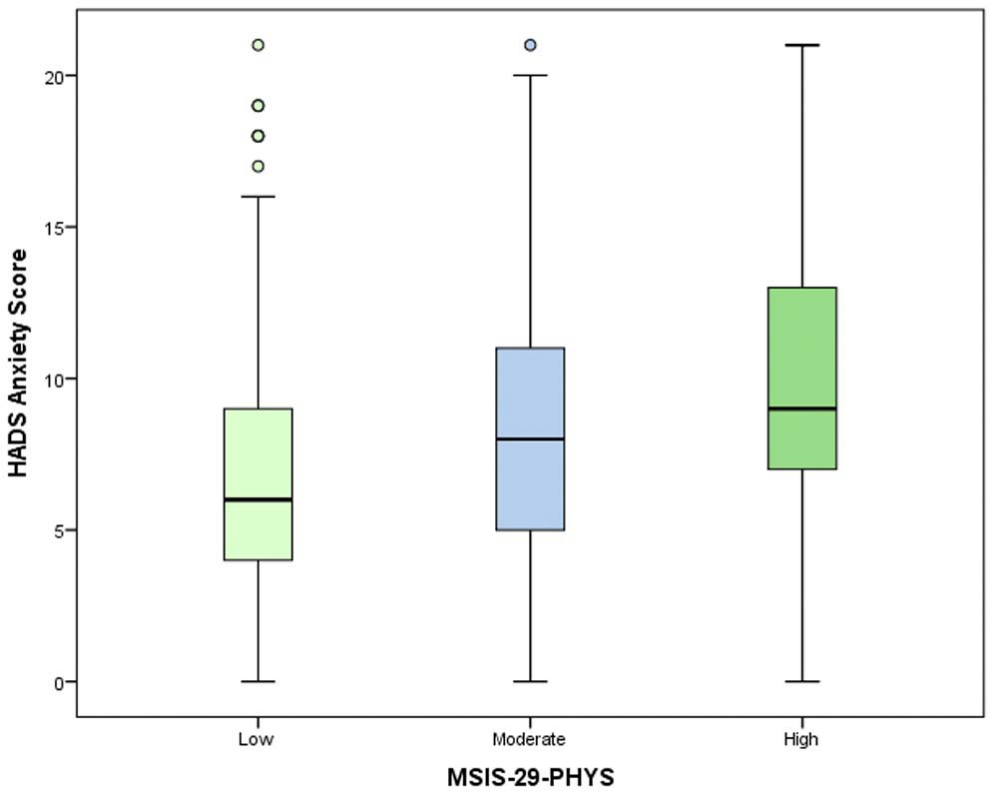
The variation in anxiety scores by physical disability. The variation in the median (and IQR of the) anxiety scores in relation to MSIS-29-PHYS scores divided into tertiles and categorised as low, moderate and high disability can be seen. The anxiety scores were highest for people in the high disability category and lowest in the low disability category (*p*<0.001).

**Figure 4:**
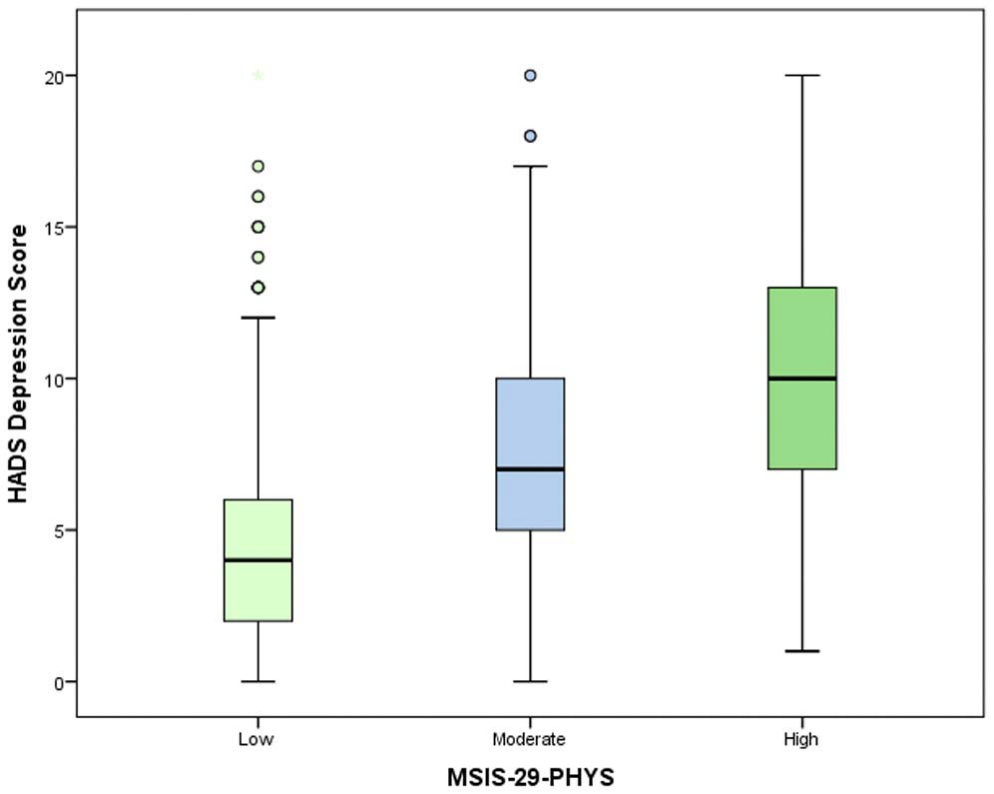
The variation in depression scores by physical disability. This shows the variation in the median (and IQR of the) depression scores in relation to MSIS-29-PHYS scores divided into tertiles and categorised as low, moderate and high disability. The depression scores were highest for people in the high disability category and lowest in the low disability category (p<0.001).

The HADS and MSIS-29-PHYS scores were divided into categories to examine frequencies and proportions (Tables S1 to S4 in the supporting information). The proportions of people reporting symptoms of anxiety increased with the MSIS-29-PHYS tertiles. Among people with low physical disability scores, the majority (62%) were not suffering from anxiety and only 1.5% were experiencing severe anxiety. However, among those with high physical disability scores only a third (33.3%) had anxiety levels in the normal category and almost a tenth (9.8%) reported severe anxiety (chi-square, *p*<0.001). A similar pattern was observed for depression. Over four fifths (82.9%) of people with low physical disability scores were not experiencing depression, and just 0.4% reported severe depression. For those with a high physical disability score, just 28.3% feel into the normal category, and 9.4% reported severe depression (chi-square, *p*<0.001). The results are illustrated in Figures 1 and 2. A similar chart displaying the relationship between the MSIS-29-PHYS and MSIS-29-PSYCH was shown in a previous article [28]. As well as differences in the frequencies of people suffering from anxiety and depression by physical disability level, differences were also found in the anxiety and depression scores. Anxiety scores were highest in respondents in the high disability category, and the pattern was repeated when depression scores were analysed. (*p*<0.001, *p*<0.001). The median and IQR values (in brackets) for anxiety by physical score in tertiles were: 6 (5) for the low, 8 (6) for the moderate and 9 (7) for the high disability groups. For depression the values were: 4 (4) for the low, 7 (5) for the moderate and 10 (6) for the high disability groups. These patterns are illustrated in Figures 3 and 4, respectively.

### Initial inter-scale measures

It has been shown that there is a moderately strong positive relationship (*rho*  = 0.6, *p*<0.001) between the physical and psychological sub-scales of the MSIS-29 [25], [28]. In beginning to explore physical disability, anxiety and depression using the MSIS-29 linked to the HADS, the relationships between the scales were assessed by comparing the scores. A weak to moderate positive relationship was found between the MSIS-29-PHYS and anxiety scores (*r* = 0.301, adjusted *r* ^2^ = 0.090, *p*<0.001) and a moderately strong positive relationship was found between the MSIS-29-PHYS and depression scores (*r* = 0.593, adjusted *r* ^2^ = 0.301, *p*<0.001). This suggested that there are differences in how physical disability impacts upon anxiety and depression, but as it was known from previous studies that mental wellbeing can vary with certain factors in a cohort such as this [27], [28], further analyses were conducted including gender, age and disease course and disease duration.

### Modelling anxiety and depression

We developed multiple regression models using the HADS anxiety or depression score as the dependent variable (DV) and using the MSIS-29-PHYS score, gender, age, disease course and disease duration as the independent variables (IVs). For anxiety, the best model included the physical score, age, male gender, disease duration and a disease course of PPMS. The model excluded female gender, RRMS and SPMS due to co-linearity (all *p*<0.001). The adjusted *R* ^2^ was 0.184; Durbin-Watson 1.984; ANOVA *F* = 161.508, df = 5, *p*<0.001. The standardised coefficients *β* were: physical score 0.398, male gender −0.076, PPMS −0.062, disease duration −0.080, and age −0.218 (all *p*<0.001). This indicates that the MSIS-29-PHYS score is the major contributor to the increase in the anxiety score, and that the other factors actually reduce the effect, with age being the most important of these (Table 2). For depression, the best model included the physical score, age, male gender, disease duration and a disease course of SPMS. The model excluded female gender, PPMS and RRMS due to co-linearity (all *p*<0.001). The adjusted *R* ^2^ was 0.378; Durbin-Watson 1.943; ANOVA *F* = 432.983, df = 5, *p*<0.001. The standardised coefficients *β* were: physical score 0.636, male gender 0.040, SPMS −0.031, disease duration −0.075, and age −0.064 (all *p*<0.001, except male gender  = 0.003, SPMS  = 0.032). This shows that, of the variables tested, MSIS-29-PHYS score is the major contributor to the increase in the depression score. With the exception of being male, the other factors in the model actually reduce the effect to a small extent (Table 3).

**Table 2:** Multiple Regression Coefficients for MSIS Physical Score for Anxiety.

Summary					
Model	R	R Square	Adjusted R Square	Std. Error of the Estimate	Durbin-Watson
Anxiety	0.420^a^	0.185	0.184	2.00474	1.984
**Coefficients**					
Model	B^b^	Std. Error^b^	Beta^c^	*t*	*p* value
Constant	4.772	0.203	-	23.476	<0.001
MSIS Physical Score	0.007	0.000	0.398	24.980	<0.001
Age	−0.016	0.001	−0.218	−11.684	<0.001
Length of Diagnosis	−0.128	0.029	−0.080	−4.355	<0.001
Gender	0.372	0.076	0.076	−4.917	<0.001
Disease Course	−0.389	0.102	−0.062	−3.806	<0.001

The multiple regression adjusted R2 for anxiety is 0.184 (*p*<0.001). The strongest relationship is with the MSIS Physical Score (Standardised Coefficient 0.389; *p*<0.001) and there was a negative relationship with Age (Standardised Coefficient −0.218; *p*<0.001).

a Predictors: (Constant), MSIS Physical Score, Age, Length of Confirmation, Gender, Disease Course.

b Unstandardised coefficient.

c Standardised coefficient.

**Table 3:** Multiple Regression Coefficients for MSIS Physical Score for Depression.

Summary					
Model	R	R Square	Adjusted R Square	Std. Error of the Estimate	Durbin-Watson
Depression	0.615^a^	0.379	0.378	1.41753	1.943
Coefficients					
Model	B^b^	Std. Error^b^	Beta^c^	*t*	*p* value
Constant	2.582	0.144	-		
MSIS Physical Score	0.009	0.000	0.636	45.676	<0.001
Age	−0.004	0.001	−0.064	−3.910	<0.001
Length of Diagnosis	−0.097	0.021	−0.075	−4.676	<0.001
Gender	−0.160	0.054	−0.40	02.992	= 0.003
Disease Course	−0.155	0.072	−0.031	−2.151	= 0.032

The multiple regression adjusted R2 for depression is 0.378 (*p*<0.001). The strongest relationship is with the MSIS Physical Score (Standardised Coefficient 0.636; *p*<0.001) with the other factors only having a relatively small, mostly negative effect.

a Predictors: (Constant), MSIS Physical Score, Age, Length of Confirmation, Gender, Disease Course.

b Unstandardised coefficient.

c Standardised coefficient.

## Discussion

### Principal Findings

This study has examined the relationships between physical disability, anxiety and depression in a large cohort of people with MS. We have found that the proportions of people experiencing anxiety and depression increase with physical disability. Among people with a low MSIS-29-PHYS score, 38% reported symptoms of anxiety, whereas among people with a high MSIS-29-PHYS score 66.7% experienced at least mild anxiety. A similar pattern was observed in the proportions suffering from depression where 17.1% with low disability and 71.7% with high disability reported at least mild symptoms. This indicates that there is a marked difference in the proportions experiencing anxiety and depression in relation to their physical disability. Furthermore, the anxiety and depression scores of the respondents were found to be higher among those reporting greater disability. The median score for anxiety was 6 in the low, and 9 in the high disability groups. An even more pronounced difference was observed in the medians for depression, with a median value of 4 for the low, and 10 for the high disability groups. So not only do larger proportions of people with greater disability experience anxiety and depression, but people who are more disabled have higher levels of these indicators of mental ill-health.

Simple linear regressions with the MSIS-29-PHYS scores as IV and depression or anxiety scores as DV showed a positive relationship; though for anxiety the model explained less than 10% of the change in scores. In previous studies [27], [28] the relationships between physical disability, anxiety and depression were observed to vary with certain factors, and by including these, we have been able to develop better models for the impact of physical disability on anxiety and depression. Using multiple regression analyses, this study found a positive relationship between physical disability and anxiety, and between physical disability and depression. Compared to the other variables included, physical disability was found to be the strongest predictor of anxiety and of depression with standardised coefficients *β* of 0.398 and 0.636 respectively. Although the overall adjusted *R* ^2^ indicated that the model explained less than 20% of the variance in anxiety, the findings confirm an earlier observation that age and anxiety can be negatively related [27], and underline the importance of ensuring that moderating factors are taken into account in assessing anxiety in people with MS so that it is not masked and underestimated. However, the models still only explain part of the variance in anxiety (<20%) and depression (<40%), which leads us to conclude that there must be other significant factors involved. Even so, these results highlight the important link between physical disability, anxiety and depression in people with MS.

### Limitations

This study was conducted on a self-selected sample of people with MS using self-reported measures of physical disability, anxiety and depression collected via the internet. This may introduce bias as this cohort may not be representative of the prevalent population of people with MS in the UK. For example, we have a lower than expected proportion of people reporting SPMS [26]. However, the mean age and gender distribution of our respondents accords with published findings, and using educational attainment as a factor that can be compared with the general UK population it was found that our cohort was of a very similar profile [26]. This needs to be recognised as a limitation but it is worth noting that the work described here is on a well characterised cohort and uses within group comparisons. In time as the MS Register builds up its data from NHS Neurology clinics, we will be able to use these data as the gold standard to validate portal data and to assess and account for bias in the self-reported responses. The MS Register has been designed on an electronic data collection model and it is possible that bias is introduced by collecting data via the internet, particularly for the technically inexperienced, elderly or socially disadvantaged [39], [40]. However, the value of remote data collection methods is increasingly being recognised in epidemiologic studies, and the evidence largely supports web and mail as being comparable media for questionnaire delivery [41). For people with MS in particular, a recent study showed that over 85% used the internet, with the majority using it as the first source of health information [42]. Another study showed that about 90% of people with MS have a personal computer and use the internet at least once a week [43]. We were only able to include some factors in our models to assess the relationship between physical disability, anxiety and depression and our models only explain part of the variance. For example, we do not currently have sufficient information on relapses or on fatigue to factor these in, and both of these have been reported to influence depression [8], [44]. Another limitation of this study is that it is cross-sectional in design and so caution is needed in inferring causation. But as the MS Register continues to collect data, in the future we will be able to carry out longitudinal studies. The Register is still in early stages of its development, and although it opens up new opportunities for novel studies, some will still need more traditional data collection methods and settings [45].

### Comparison with Prior Work

The relationship between chronic illness and mental wellbeing has been studied in a range of conditions [46] and people with MS have been found to experience high levels of anxiety and depression [1], [2], [27], often higher than among people with other chronic conditions [47]. Various studies have looked at physical disability in relation to depression among people with MS and these have produced mixed results, with some authors reporting an association and some not finding one [8]–[13]. This is the only study, however, that used the MSIS-29 linked to the HADS scale and at least some of the variations might be due to differing instruments, as well as to differences in size and characteristics of the samples groups. Fewer studies have been conducted on anxiety in people with MS, but it has been observed that anxiety is also of high prevalence and that it can affect the family members as well [10], [11], [27], [48]. It has also been found that people suffering from mental ill-health are more likely to have problems in adhering to disease-modifying medications [49]. It is a matter for concern that people with MS have not in the past been adequately screened for anxiety and depression and, consequently, these conditions have been under-treated [6], [50]. While this is not always the case, it is acknowledged that further studies are needed on the use and efficacy of antidepressant and anxiolytic medications among people with MS, as well as other treatments and therapies for these conditions [51]. This is an area that we will be able to address with data from the MS Register in the future as we have begun collecting medications information. Lack of social support is another factor that has been linked to depression among people with MS [52]. This, and the likely influences of co-morbidities, fatigue and relapses on anxiety and depression, are other issues we will be able to study due course. The aetiology of anxiety and depression in people with MS is the subject of much debate as to whether the main causes are organic or reactive in origin [1], [12], [13]. This is not something that we can examine with our data, but, whatever the causes of mental ill-health, it is imperative that people are given the treatment and care they need so that they and their families can have the best possible quality of life.

## Conclusions

This novel study using >4500 responses to the MSIS-29 and HADS scales has indicated that there is a positive relationship between physical disability and anxiety and depression. It has shown that physical disability impacts on anxiety and depression to differing extents, and that the effects vary with gender, age, disease course and disease duration. By including these moderating factors in separate multiple regression models, we were able to show that physical disability is a predictor of anxiety and depression, and that other factors may actually mask the extent of this effect. Our models were able to account for some of the variance in anxiety and depression, but clearly, there must be other factors at work. These may include fatigue and relapses in relation to MS, the presence of other medical conditions, or the availability of social support [8], [44], [52]. Whether the causes of anxiety and depression are reactive, organic or a combination, it is essential that mental wellbeing is given due attention in caring for people with MS so that all their health needs can be met. We acknowledge the limitations of the study design in the use of self-reported data collected via the internet and in not being able to incorporate all factors that could be influential.

## Supporting Information

Table S1 **Proportions and frequencies: MSIS-29-PHYS.** The proportions and frequencies of respondents in the low, moderate and high tertiles of the MSIS-29-PHYS, stratified by age, gender and disease course are shown.
(DOCX)

Table S2 **Proportions and frequencies: MSIS-29-PSYCH.** The proportions and frequencies of respondents in the low, moderate and high tertiles of the MSIS-29-PSYCH, stratified by age, gender and disease course are shown.
(DOCX)

Table S3 **Proportions and frequencies: HADS-A.** This shows the proportions and frequencies of respondents reporting normal (scores up to 7), mild (8–10), moderate (11–15) and severe anxiety (over 15), stratified by age, gender and disease course.
(DOCX)

Table S4 **Proportions and frequencies: HADS-D.** This shows the proportions and frequencies of respondents reporting normal (scores up to 7), mild (8–10), moderate (11–15) and severe depression (over 15), stratified by age, gender and disease course.
(DOCX)
