# Supplementary material for: Physical Disability, Anxiety and Depression in People with MS: An Internet-Based Survey via the UK MS Register
Source: PLoS One. 2014 Aug 25;9(8):e104604. doi: 10.1371/journal.pone.0104604 (PMC4143231; doi:10.1371/journal.pone.0104604)
Supplement: Table S1 — Proportions and frequencies: MSIS-29-PHYS. The proportions and frequencies of respondents in the low, moderate and high tertiles of the MSIS-29-PHYS, stratified by age, gender and disease course are shown. (DOCX) [file pone.0104604.s001.docx]

**Supplementary Table A.**

| Age Band | Gender | Disease Course | | MSIS Physical Score Tertiles | | | Total |
| --- | --- | --- | --- | --- | --- | --- | --- |
|  |  |  |  | Low | Moderate | High |  |
| <= 39 | Male | Primary progressive | No. of Cases | 0 | 6 | 5 | 11 |
|  |  |  | % within Disease Course | 0.0% | 54.5% | 45.5% | 100% |
|  |  | Relapsing-remitting | No. of Cases | 70 | 39 | 22 | 131 |
|  |  |  | % within Disease Course | 53.4% | 29.8% | 16.8% | 100% |
|  |  | Secondary progressive | No. of Cases | 0 | 1 | 2 | 3 |
|  |  |  | % within Disease Course | 0.0% | 33.3% | 66.7% | 100% |
|  |  | Don't know | No. of Cases | 11 | 4 | 2 | 17 |
|  |  |  | % within Disease Course | 64.7% | 23.5% | 11.8% | 100% |
|  |  | Total | No. of Cases | 81 | 50 | 31 | 162 |
|  |  |  | % within Disease Course | 50.0% | 30.9% | 19.1% | 100% |
|  | Female | Primary progressive | No. of Cases | 6 | 6 | 5 | 17 |
|  |  |  | % within Disease Course | 35.3% | 35.3% | 29.4% | 100% |
|  |  | Relapsing-remitting | No. of Cases | 266 | 156 | 109 | 531 |
|  |  |  | % within Disease Course | 50.1% | 29.4% | 20.5% | 100% |
|  |  | Secondary progressive | No. of Cases | 0 | 2 | 2 | 4 |
|  |  |  | % within Disease Course | 0.0% | 50.0% | 50% | 100% |
|  |  | Don't know | No. of Cases | 15 | 12 | 12 | 39 |
|  |  |  | % within Disease Course | 38.5% | 30.8% | 30.8% | 100% |
|  |  | Total | No. of Cases | 287 | 176 | 128 | 591 |
|  |  |  | % within Disease Course | 48.6% | 29.8% | 21.7% | 100% |
|  | Total | Primary progressive | No. of Cases | 6 | 12 | 10 | 28 |
|  |  |  | % within Disease Course | 21.4% | 42.9% | 35.7% | 100% |
|  |  | Relapsing-remitting | No. of Cases | 336 | 195 | 131 | 662 |
|  |  |  | % within Disease Course | 50.8% | 29.5% | 19.8% | 100% |
|  |  | Secondary progressive | No. of Cases | 0 | 3 | 4 | 7 |
|  |  |  | % within Disease Course | 0.0% | 42.9% | 57.1% | 100% |
|  |  | Don't know | No. of Cases | 26 | 16 | 14 | 56 |
|  |  |  | % within Disease Course | 46.4% | 28.6% | 25% | 100% |
|  |  | Total | No. of Cases | 368 | 226 | 159 | 753 |
|  |  |  | % within Disease Course | 48.9% | 30.0% | 21.1% | 100% |
| 40 to 49 | Male | Primary progressive | No. of Cases | 7 | 24 | 24 | 55 |
|  |  |  | % within Disease Course | 12.7% | 43.6% | 43.6% | 100% |
|  |  | Relapsing-remitting | No. of Cases | 85 | 84 | 59 | 228 |
|  |  |  | % within Disease Course | 37.3% | 36.8% | 25.9% | 100% |
|  |  | Secondary progressive | No. of Cases | 0 | 8 | 15 | 23 |
|  |  |  | % within Disease Course | 0.0% | 34.8% | 65.2% | 100% |
|  |  | Don't know | No. of Cases | 5 | 11 | 12 | 28 |
|  |  |  | % within Disease Course | 17.9% | 39.3% | 42.9% | 100% |
|  |  | Total | No. of Cases | 97 | 127 | 110 | 334 |
|  |  |  | % within Disease Course | 29.0% | 38.0% | 32.9% | 100% |
|  | Female | Primary progressive | No. of Cases | 8 | 21 | 32 | 61 |
|  |  |  | % within Disease Course | 13.1% | 34.4% | 52.5% | 100% |
|  |  | Relapsing-remitting | No. of Cases | 291 | 268 | 174 | 733 |
|  |  |  | % within Disease Course | 39.7% | 36.6% | 23.7% | 100% |
|  |  | Secondary progressive | No. of Cases | 2 | 16 | 21 | 39 |
|  |  |  | % within Disease Course | 5.1% | 41.0% | 53.8% | 100% |
|  |  | Don't know | No. of Cases | 41 | 36 | 31 | 108 |
|  |  |  | % within Disease Course | 38.0% | 33.3% | 28.7% | 100% |
|  |  | Total | No. of Cases | 342 | 341 | 258 | 941 |
|  |  |  | % within Disease Course | 36.3% | 36.2% | 27.4% | 100% |
|  | Total | Primary progressive | No. of Cases | 15 | 45 | 56 | 116 |
|  |  |  | % within Disease Course | 12.9% | 38.8% | 48.3% | 100% |
|  |  | Relapsing-remitting | No. of Cases | 376 | 352 | 233 | 961 |
|  |  |  | % within Disease Course | 39.1% | 36.6% | 24.2% | 100% |
|  |  | Secondary progressive | No. of Cases | 2 | 24 | 36 | 62 |
|  |  |  | % within Disease Course | 3.2% | 38.7% | 58.1% | 100% |
|  |  | Don't know | No. of Cases | 46 | 47 | 43 | 136 |
|  |  |  | % within Disease Course | 33.8% | 34.6% | 31.6% | 100% |
|  |  | Total | No. of Cases | 439 | 468 | 368 | 1275 |
|  |  |  | % within Disease Course | 34.4% | 36.7% | 28.9% | 100% |
| 50 to 59 | Male | Primary progressive | No. of Cases | 14 | 32 | 48 | 94 |
|  |  |  | % within Disease Course | 14.9% | 34.0% | 51.1% | 100% |
|  |  | Relapsing-remitting | No. of Cases | 40 | 65 | 75 | 180 |
|  |  |  | % within Disease Course | 22.2% | 36.1% | 41.7% | 100% |
|  |  | Secondary progressive | No. of Cases | 2 | 25 | 28 | 55 |
|  |  |  | % within Disease Course | 3.6% | 45.5% | 50.9% | 100% |
|  |  | Don't know | No. of Cases | 16 | 24 | 19 | 59 |
|  |  |  | % within Disease Course | 27.1% | 40.7% | 32.2% | 100% |
|  |  | Total | No. of Cases | 72 | 146 | 170 | 388 |
|  |  |  | % within Disease Course | 18.6% | 37.6% | 43.8% | 100% |
|  | Female | Primary progressive | No. of Cases | 15 | 55 | 59 | 129 |
|  |  |  | % within Disease Course | 11.6% | 42.6% | 45.7% | 100% |
|  |  | Relapsing-remitting | No. of Cases | 174 | 245 | 177 | 596 |
|  |  |  | % within Disease Course | 29.2% | 41.1% | 29.7% | 100% |
|  |  | Secondary progressive | No. of Cases | 5 | 24 | 46 | 75 |
|  |  |  | % within Disease Course | 6.7% | 32.0% | 61.3% | 100% |
|  |  | Don't know | No. of Cases | 49 | 65 | 48 | 162 |
|  |  |  | % within Disease Course | 30.2% | 40.1% | 29.6% | 100% |
|  |  | Total | No. of Cases | 243 | 389 | 330 | 962 |
|  |  |  | % within Disease Course | 25.3% | 40.4% | 34.3% | 100% |
|  | Total | Primary progressive | No. of Cases | 29 | 87 | 107 | 223 |
|  |  |  | % within Disease Course | 13.0% | 39.0% | 48% | 100% |
|  |  | Relapsing-remitting | No. of Cases | 214 | 310 | 252 | 776 |
|  |  |  | % within Disease Course | 27.6% | 39.9% | 32.5% | 100% |
|  |  | Secondary progressive | No. of Cases | 7 | 49 | 74 | 130 |
|  |  |  | % within Disease Course | 5.4% | 37.7% | 56.9% | 100% |
|  |  | Don't know | No. of Cases | 65 | 89 | 67 | 221 |
|  |  |  | % within Disease Course | 29.4% | 40.3% | 30.3% | 100% |
|  |  | Total | No. of Cases | 315 | 535 | 500 | 1350 |
|  |  |  | % within Disease Course | 23.3% | 39.6% | 37.0% | 100% |
| 60 to 69 | Male | Primary progressive | No. of Cases | 17 | 48 | 56 | 121 |
|  |  |  | % within Disease Course | 14.0% | 39.7% | 46.3% | 100% |
|  |  | Relapsing-remitting | No. of Cases | 26 | 42 | 31 | 99 |
|  |  |  | % within Disease Course | 26.3% | 42.4% | 31.3% | 100% |
|  |  | Secondary progressive | No. of Cases | 4 | 20 | 25 | 49 |
|  |  |  | % within Disease Course | 8.2% | 40.8% | 51% | 100% |
|  |  | Don't know | No. of Cases | 17 | 22 | 16 | 55 |
|  |  |  | % within Disease Course | 30.9% | 40.0% | 29.1% | 100% |
|  |  | Total | No. of Cases | 64 | 132 | 128 | 324 |
|  |  |  | % within Disease Course | 19.8% | 40.7% | 39.5% | 100% |
|  | Female | Primary progressive | No. of Cases | 12 | 48 | 43 | 103 |
|  |  |  | % within Disease Course | 11.7% | 46.6% | 41.7% | 100% |
|  |  | Relapsing-remitting | No. of Cases | 64 | 79 | 67 | 210 |
|  |  |  | % within Disease Course | 30.5% | 37.6% | 31.9% | 100% |
|  |  | Secondary progressive | No. of Cases | 5 | 43 | 31 | 79 |
|  |  |  | % within Disease Course | 6.3% | 54.4% | 39.2% | 100% |
|  |  | Don't know | No. of Cases | 38 | 50 | 50 | 138 |
|  |  |  | % within Disease Course | 27.5% | 36.2% | 36.2% | 100% |
|  |  | Total | No. of Cases | 119 | 220 | 191 | 530 |
|  |  |  | % within Disease Course | 22.5% | 41.5% | 36.0% | 100% |
|  | Total | Primary progressive | No. of Cases | 29 | 96 | 99 | 224 |
|  |  |  | % within Disease Course | 12.9% | 42.9% | 44.2% | 100% |
|  |  | Relapsing-remitting | No. of Cases | 90 | 121 | 98 | 309 |
|  |  |  | % within Disease Course | 29.1% | 39.2% | 31.7% | 100% |
|  |  | Secondary progressive | No. of Cases | 9 | 63 | 56 | 128 |
|  |  |  | % within Disease Course | 7.0% | 49.2% | 43.8% | 100% |
|  |  | Don't know | No. of Cases | 55 | 72 | 66 | 193 |
|  |  |  | % within Disease Course | 28.5% | 37.3% | 34.2% | 100% |
|  |  | Total | No. of Cases | 183 | 352 | 319 | 854 |
|  |  |  | % within Disease Course | 21.4% | 41.2% | 37.4% | 100% |
| >=70 | Male | Primary progressive | No. of Cases | 3 | 15 | 9 | 27 |
|  |  |  | % within Disease Course | 11.1% | 55.6% | 33.3% | 100% |
|  |  | Relapsing-remitting | No. of Cases | 2 | 5 | 3 | 10 |
|  |  |  | % within Disease Course | 20.0% | 50.0% | 30% | 100% |
|  |  | Secondary progressive | No. of Cases | 0 | 3 | 6 | 9 |
|  |  |  | % within Disease Course | 0.0% | 33.3% | 66.7% | 100% |
|  |  | Don't know | No. of Cases | 5 | 11 | 5 | 21 |
|  |  |  | % within Disease Course | 23.8% | 52.4% | 23.8% | 100% |
|  |  | Total | No. of Cases | 10 | 34 | 23 | 67 |
|  |  |  | % within Disease Course | 14.9% | 50.7% | 34.3% | 100% |
|  | Female | Primary progressive | No. of Cases | 4 | 14 | 14 | 32 |
|  |  |  | % within Disease Course | 12.5% | 43.8% | 43.8% | 100% |
|  |  | Relapsing-remitting | No. of Cases | 2 | 7 | 10 | 19 |
|  |  |  | % within Disease Course | 10.5% | 36.8% | 52.6% | 100% |
|  |  | Secondary progressive | No. of Cases | 3 | 10 | 10 | 23 |
|  |  |  | % within Disease Course | 13.0% | 43.5% | 43.5% | 100% |
|  |  | Don't know | No. of Cases | 10 | 12 | 9 | 31 |
|  |  |  | % within Disease Course | 32.3% | 38.7% | 29% | 100% |
|  |  | Total | No. of Cases | 19 | 43 | 43 | 105 |
|  |  |  | % within Disease Course | 18.1% | 41.0% | 41.0% | 100% |
|  | Total | Primary progressive | No. of Cases | 7 | 29 | 23 | 59 |
|  |  |  | % within Disease Course | 11.9% | 49.2% | 39% | 100% |
|  |  | Relapsing-remitting | No. of Cases | 4 | 12 | 13 | 29 |
|  |  |  | % within Disease Course | 13.8% | 41.4% | 44.8% | 100% |
|  |  | Secondary progressive | No. of Cases | 3 | 13 | 16 | 32 |
|  |  |  | % within Disease Course | 9.4% | 40.6% | 50% | 100% |
|  |  | Don't know | No. of Cases | 15 | 23 | 14 | 52 |
|  |  |  | % within Disease Course | 28.8% | 44.2% | 26.9% | 100% |
|  |  | Total | No. of Cases | 29 | 77 | 66 | 172 |
|  |  |  | % within Disease Course | 16.9% | 44.8% | 38.4% | 100% |
| All Ages | Male | Primary progressive | No. of Cases | 41 | 125 | 142 | 308 |
|  |  |  | % within Disease Course | 13.3% | 40.6% | 46.1% | 100% |
|  |  | Relapsing-remitting | No. of Cases | 223 | 235 | 190 | 648 |
|  |  |  | % within Disease Course | 34.4% | 36.3% | 29.3% | 100% |
|  |  | Secondary progressive | No. of Cases | 6 | 57 | 76 | 139 |
|  |  |  | % within Disease Course | 4.3% | 41.0% | 54.7% | 100% |
|  |  | Don't know | No. of Cases | 54 | 72 | 54 | 180 |
|  |  |  | % within Disease Course | 30.0% | 40.0% | 30% | 100% |
|  |  | Total | No. of Cases | 324 | 489 | 462 | 1275 |
|  |  |  | % within Disease Course | 25.4% | 38.4% | 36.2% | 100% |
|  | Female | Primary progressive | No. of Cases | 45 | 144 | 153 | 342 |
|  |  |  | % within Disease Course | 13.2% | 42.1% | 44.7% | 100% |
|  |  | Relapsing-remitting | No. of Cases | 797 | 755 | 537 | 2089 |
|  |  |  | % within Disease Course | 38.2% | 36.1% | 25.7% | 100% |
|  |  | Secondary progressive | No. of Cases | 15 | 95 | 110 | 220 |
|  |  |  | % within Disease Course | 6.8% | 43.2% | 50% | 100% |
|  |  | Don't know | No. of Cases | 153 | 175 | 150 | 478 |
|  |  |  | % within Disease Course | 32.0% | 36.6% | 31.4% | 100% |
|  |  | Total | No. of Cases | 1010 | 1169 | 950 | 3129 |
|  |  |  | % within Disease Course | 32.3% | 37.4% | 30.4% | 100% |
|  | Total | Primary progressive | No. of Cases | 86 | 269 | 295 | 650 |
|  |  |  | % within Disease Course | 13.2% | 41.4% | 45.4% | 100% |
|  |  | Relapsing-remitting | No. of Cases | 1020 | 990 | 727 | 2737 |
|  |  |  | % within Disease Course | 37.3% | 36.2% | 26.6% | 100% |
|  |  | Secondary progressive | No. of Cases | 21 | 152 | 186 | 359 |
|  |  |  | % within Disease Course | 5.8% | 42.3% | 51.8% | 100% |
|  |  | Don't know | No. of Cases | 207 | 247 | 204 | 658 |
|  |  |  | % within Disease Course | 31.5% | 37.5% | 31% | 100% |
|  |  | Total | No. of Cases | 1334 | 1658 | 1412 | 4404 |
|  |  |  | % within Disease Course | 30.3% | 37.6% | 32.1% | 100% |
