# Supplementary material for: Physical Disability, Anxiety and Depression in People with MS: An Internet-Based Survey via the UK MS Register
Source: PLoS One. 2014 Aug 25;9(8):e104604. doi: 10.1371/journal.pone.0104604 (PMC4143231; doi:10.1371/journal.pone.0104604)
Supplement: Table S2 — Proportions and frequencies: MSIS-29-PSYCH. The proportions and frequencies of respondents in the low, moderate and high tertiles of the MSIS-29-PSYCH, stratified by age, gender and disease course are shown. (DOCX) [file pone.0104604.s002.docx]

**Supplementary Table B.**

| Age Band | Gender | Disease Course | | MSIS Psychological Tertiles | | | Total |
| --- | --- | --- | --- | --- | --- | --- | --- |
|  |  |  |  | Low | Moderate | High |  |
| <= 39 | Male | Primary progressive | No. of Cases | 0 | 6 | 5 | 11 |
|  |  |  | % within Disease Course | 0.0% | 54.5% | 45.5% | 100% |
|  |  | Relapsing-remitting | No. of Cases | 53 | 49 | 29 | 131 |
|  |  |  | % within Disease Course | 40.5% | 37.4% | 22.1% | 100% |
|  |  | Secondary progressive | No. of Cases | 0 | 2 | 1 | 3 |
|  |  |  | % within Disease Course | 0.0% | 66.7% | 33.3% | 100% |
|  |  | Don't know | No. of Cases | 6 | 7 | 4 | 17 |
|  |  |  | % within Disease Course | 35.3% | 41.2% | 23.5% | 100% |
|  |  | Total | No. of Cases | 59 | 64 | 39 | 162 |
|  |  |  | % within Disease Course | 36.4% | 39.5% | 24.1% | 100% |
|  | Female | Primary progressive | No. of Cases | 3 | 8 | 6 | 17 |
|  |  |  | % within Disease Course | 17.6% | 47.1% | 35.3% | 100% |
|  |  | Relapsing-remitting | No. of Cases | 203 | 212 | 116 | 531 |
|  |  |  | % within Disease Course | 38.2% | 39.9% | 21.8% | 100% |
|  |  | Secondary progressive | No. of Cases | 2 | 2 | 0 | 4 |
|  |  |  | % within Disease Course | 50.0% | 50.0% | 0.0% | 100% |
|  |  | Don't know | No. of Cases | 10 | 15 | 14 | 39 |
|  |  |  | % within Disease Course | 25.6% | 38.5% | 35.9% | 100% |
|  |  | Total | No. of Cases | 218 | 237 | 136 | 591 |
|  |  |  | % within Disease Course | 36.9% | 40.1% | 23.0% | 100% |
|  | Total | Primary progressive | No. of Cases | 3 | 14 | 11 | 28 |
|  |  |  | % within Disease Course | 10.7% | 50.0% | 39.3% | 100% |
|  |  | Relapsing-remitting | No. of Cases | 256 | 261 | 145 | 662 |
|  |  |  | % within Disease Course | 38.7% | 39.4% | 21.9% | 100% |
|  |  | Secondary progressive | No. of Cases | 2 | 4 | 1 | 7 |
|  |  |  | % within Disease Course | 28.6% | 57.1% | 14.3% | 100% |
|  |  | Don't know | No. of Cases | 16 | 22 | 18 | 56 |
|  |  |  | % within Disease Course | 28.6% | 39.3% | 32.1% | 100% |
|  |  | Total | No. of Cases | 277 | 301 | 175 | 753 |
|  |  |  | % within Disease Course | 36.8% | 40.0% | 23.2% | 100% |
| 40 to 49 | Male | Primary progressive | No. of Cases | 12 | 26 | 17 | 55 |
|  |  |  | % within Disease Course | 21.8% | 47.3% | 30.9% | 100% |
|  |  | Relapsing-remitting | No. of Cases | 84 | 99 | 45 | 228 |
|  |  |  | % within Disease Course | 36.8% | 43.4% | 19.7% | 100% |
|  |  | Secondary progressive | No. of Cases | 5 | 9 | 9 | 23 |
|  |  |  | % within Disease Course | 21.7% | 39.1% | 39.1% | 100% |
|  |  | Don't know | No. of Cases | 7 | 11 | 10 | 28 |
|  |  |  | % within Disease Course | 25.0% | 39.3% | 35.7% | 100% |
|  |  | Total | No. of Cases | 108 | 145 | 81 | 334 |
|  |  |  | % within Disease Course | 32.3% | 43.4% | 24.3% | 100% |
|  | Female | Primary progressive | No. of Cases | 17 | 23 | 21 | 61 |
|  |  |  | % within Disease Course | 27.9% | 37.7% | 34.4% | 100% |
|  |  | Relapsing-remitting | No. of Cases | 293 | 287 | 153 | 733 |
|  |  |  | % within Disease Course | 40.0% | 39.2% | 20.9% | 100% |
|  |  | Secondary progressive | No. of Cases | 5 | 22 | 12 | 39 |
|  |  |  | % within Disease Course | 12.8% | 56.4% | 30.8% | 100% |
|  |  | Don't know | No. of Cases | 40 | 45 | 23 | 108 |
|  |  |  | % within Disease Course | 37.0% | 41.7% | 21.3% | 100% |
|  |  | Total | No. of Cases | 355 | 377 | 209 | 941 |
|  |  |  | % within Disease Course | 37.7% | 40.1% | 22.2% | 100% |
|  | Total | Primary progressive | No. of Cases | 29 | 49 | 38 | 116 |
|  |  |  | % within Disease Course | 25.0% | 42.2% | 32.8% | 100% |
|  |  | Relapsing-remitting | No. of Cases | 377 | 386 | 198 | 961 |
|  |  |  | % within Disease Course | 39.2% | 40.2% | 20.6% | 100% |
|  |  | Secondary progressive | No. of Cases | 10 | 31 | 21 | 62 |
|  |  |  | % within Disease Course | 16.1% | 50.0% | 33.9% | 100% |
|  |  | Don't know | No. of Cases | 47 | 56 | 33 | 136 |
|  |  |  | % within Disease Course | 34.6% | 41.2% | 24.3% | 100% |
|  |  | Total | No. of Cases | 463 | 522 | 290 | 1275 |
|  |  |  | % within Disease Course | 36.3% | 40.9% | 22.7% | 100% |
| 50 to 59 | Male | Primary progressive | No. of Cases | 40 | 33 | 21 | 94 |
|  |  |  | % within Disease Course | 42.6% | 35.1% | 22.3% | 100% |
|  |  | Relapsing-remitting | No. of Cases | 65 | 72 | 43 | 180 |
|  |  |  | % within Disease Course | 36.1% | 40.0% | 23.9% | 100% |
|  |  | Secondary progressive | No. of Cases | 14 | 28 | 13 | 55 |
|  |  |  | % within Disease Course | 25.5% | 50.9% | 23.6% | 100% |
|  |  | Don't know | No. of Cases | 20 | 29 | 10 | 59 |
|  |  |  | % within Disease Course | 33.9% | 49.2% | 16.9% | 100% |
|  |  | Total | No. of Cases | 139 | 162 | 87 | 388 |
|  |  |  | % within Disease Course | 35.8% | 41.8% | 22.4% | 100% |
|  | Female | Primary progressive | No. of Cases | 41 | 59 | 29 | 129 |
|  |  |  | % within Disease Course | 31.8% | 45.7% | 22.5% | 100% |
|  |  | Relapsing-remitting | No. of Cases | 246 | 246 | 104 | 596 |
|  |  |  | % within Disease Course | 41.3% | 41.3% | 17.4% | 100% |
|  |  | Secondary progressive | No. of Cases | 22 | 31 | 22 | 75 |
|  |  |  | % within Disease Course | 29.3% | 41.3% | 29.3% | 100% |
|  |  | Don't know | No. of Cases | 69 | 63 | 30 | 162 |
|  |  |  | % within Disease Course | 42.6% | 38.9% | 18.5% | 100% |
|  |  | Total | No. of Cases | 378 | 399 | 185 | 962 |
|  |  |  | % within Disease Course | 39.3% | 41.5% | 19.2% | 100% |
|  | Total | Primary progressive | No. of Cases | 81 | 92 | 50 | 223 |
|  |  |  | % within Disease Course | 36.3% | 41.3% | 22.4% | 100% |
|  |  | Relapsing-remitting | No. of Cases | 311 | 318 | 147 | 776 |
|  |  |  | % within Disease Course | 40.1% | 41.0% | 18.9% | 100% |
|  |  | Secondary progressive | No. of Cases | 36 | 59 | 35 | 130 |
|  |  |  | % within Disease Course | 27.7% | 45.4% | 26.9% | 100% |
|  |  | Don't know | No. of Cases | 89 | 92 | 40 | 221 |
|  |  |  | % within Disease Course | 40.3% | 41.6% | 18.1% | 100% |
|  |  | Total | No. of Cases | 517 | 561 | 272 | 1350 |
|  |  |  | % within Disease Course | 38.3% | 41.6% | 20.1% | 100% |
| 60 to 69 | Male | Primary progressive | No. of Cases | 55 | 51 | 15 | 121 |
|  |  |  | % within Disease Course | 45.5% | 42.1% | 12.4% | 100% |
|  |  | Relapsing-remitting | No. of Cases | 51 | 36 | 12 | 99 |
|  |  |  | % within Disease Course | 51.5% | 36.4% | 12.1% | 100% |
|  |  | Secondary progressive | No. of Cases | 23 | 17 | 9 | 49 |
|  |  |  | % within Disease Course | 46.9% | 34.7% | 18.4% | 100% |
|  |  | Don't know | No. of Cases | 31 | 18 | 6 | 55 |
|  |  |  | % within Disease Course | 56.4% | 32.7% | 10.9% | 100% |
|  |  | Total | No. of Cases | 160 | 122 | 42 | 324 |
|  |  |  | % within Disease Course | 49.4% | 37.7% | 13.0% | 100% |
|  | Female | Primary progressive | No. of Cases | 55 | 43 | 5 | 103 |
|  |  |  | % within Disease Course | 53.4% | 41.7% | 4.9% | 100% |
|  |  | Relapsing-remitting | No. of Cases | 106 | 81 | 23 | 210 |
|  |  |  | % within Disease Course | 50.5% | 38.6% | 11.0% | 100% |
|  |  | Secondary progressive | No. of Cases | 36 | 37 | 6 | 79 |
|  |  |  | % within Disease Course | 45.6% | 46.8% | 7.6% | 100% |
|  |  | Don't know | No. of Cases | 54 | 59 | 25 | 138 |
|  |  |  | % within Disease Course | 39.1% | 42.8% | 18.1% | 100% |
|  |  | Total | No. of Cases | 251 | 220 | 59 | 530 |
|  |  |  | % within Disease Course | 47.4% | 41.5% | 11.1% | 100% |
|  | Total | Primary progressive | No. of Cases | 110 | 94 | 20 | 224 |
|  |  |  | % within Disease Course | 49.1% | 42.0% | 8.9% | 100% |
|  |  | Relapsing-remitting | No. of Cases | 157 | 117 | 35 | 309 |
|  |  |  | % within Disease Course | 50.8% | 37.9% | 11.3% | 100% |
|  |  | Secondary progressive | No. of Cases | 59 | 54 | 15 | 128 |
|  |  |  | % within Disease Course | 46.1% | 42.2% | 11.7% | 100% |
|  |  | Don't know | No. of Cases | 85 | 77 | 31 | 193 |
|  |  |  | % within Disease Course | 44.0% | 39.9% | 16.1% | 100% |
|  |  | Total | No. of Cases | 411 | 342 | 101 | 854 |
|  |  |  | % within Disease Course | 48.1% | 40.0% | 11.8% | 100% |
| >= 70 | Male | Primary progressive | No. of Cases | 18 | 7 | 2 | 27 |
|  |  |  | % within Disease Course | 66.7% | 25.9% | 7.4% | 100% |
|  |  | Relapsing-remitting | No. of Cases | 5 | 4 | 1 | 10 |
|  |  |  | % within Disease Course | 50.0% | 40.0% | 10.0% | 100% |
|  |  | Secondary progressive | No. of Cases | 3 | 5 | 1 | 9 |
|  |  |  | % within Disease Course | 33.3% | 55.6% | 11.1% | 100% |
|  |  | Don't know | No. of Cases | 13 | 7 | 1 | 21 |
|  |  |  | % within Disease Course | 61.9% | 33.3% | 4.8% | 100% |
|  |  | Total | No. of Cases | 39 | 23 | 5 | 67 |
|  |  |  | % within Disease Course | 58.2% | 34.3% | 7.5% | 100% |
|  | Female | Primary progressive | No. of Cases | 21 | 7 | 4 | 32 |
|  |  |  | % within Disease Course | 65.6% | 21.9% | 12.5% | 100% |
|  |  | Relapsing-remitting | No. of Cases | 8 | 7 | 4 | 19 |
|  |  |  | % within Disease Course | 42.1% | 36.8% | 21.1% | 100% |
|  |  | Secondary progressive | No. of Cases | 13 | 8 | 2 | 23 |
|  |  |  | % within Disease Course | 56.5% | 34.8% | 8.7% | 100% |
|  |  | Don't know | No. of Cases | 22 | 7 | 2 | 31 |
|  |  |  | % within Disease Course | 71.0% | 22.6% | 6.5% | 100% |
|  |  | Total | No. of Cases | 64 | 29 | 12 | 105 |
|  |  |  | % within Disease Course | 61.0% | 27.6% | 11.4% | 100% |
|  | Total | Primary progressive | No. of Cases | 39 | 14 | 6 | 59 |
|  |  |  | % within Disease Course | 66.1% | 23.7% | 10.2% | 100% |
|  |  | Relapsing-remitting | No. of Cases | 13 | 11 | 5 | 29 |
|  |  |  | % within Disease Course | 44.8% | 37.9% | 17.2% | 100% |
|  |  | Secondary progressive | No. of Cases | 16 | 13 | 3 | 32 |
|  |  |  | % within Disease Course | 50.0% | 40.6% | 9.4% | 100% |
|  |  | Don't know | No. of Cases | 35 | 14 | 3 | 52 |
|  |  |  | % within Disease Course | 67.3% | 26.9% | 5.8% | 100% |
|  |  | Total | No. of Cases | 103 | 52 | 17 | 172 |
|  |  |  | % within Disease Course | 59.9% | 30.2% | 9.9% | 100% |
| All Ages | Male | Primary progressive | No. of Cases | 125 | 123 | 60 | 308 |
|  |  |  | % within Disease Course | 40.6% | 39.9% | 19.5% | 100% |
|  |  | Relapsing-remitting | No. of Cases | 258 | 260 | 130 | 648 |
|  |  |  | % within Disease Course | 39.8% | 40.1% | 20.1% | 100% |
|  |  | Secondary progressive | No. of Cases | 45 | 61 | 33 | 139 |
|  |  |  | % within Disease Course | 32.4% | 43.9% | 23.7% | 100% |
|  |  | Don't know | No. of Cases | 77 | 72 | 31 | 180 |
|  |  |  | % within Disease Course | 42.8% | 40.0% | 17.2% | 100% |
|  |  | Total | No. of Cases | 505 | 516 | 254 | 1275 |
|  |  |  | % within Disease Course | 39.6% | 40.5% | 19.9% | 100% |
|  | Female | Primary progressive | No. of Cases | 137 | 140 | 65 | 342 |
|  |  |  | % within Disease Course | 40.1% | 40.9% | 19.0% | 100% |
|  |  | Relapsing-remitting | No. of Cases | 856 | 833 | 400 | 2089 |
|  |  |  | % within Disease Course | 41.0% | 39.9% | 19.1% | 100% |
|  |  | Secondary progressive | No. of Cases | 78 | 100 | 42 | 220 |
|  |  |  | % within Disease Course | 35.5% | 45.5% | 19.1% | 100% |
|  |  | Don't know | No. of Cases | 195 | 189 | 94 | 478 |
|  |  |  | % within Disease Course | 40.8% | 39.5% | 19.7% | 100% |
|  |  | Total | No. of Cases | 1266 | 1262 | 601 | 3129 |
|  |  |  | % within Disease Course | 40.5% | 40.3% | 19.2% | 100% |
|  | Total | Primary progressive | No. of Cases | 262 | 263 | 125 | 650 |
|  |  |  | % within Disease Course | 40.3% | 40.5% | 19.2% | 100% |
|  |  | Relapsing-remitting | No. of Cases | 1114 | 1093 | 530 | 2737 |
|  |  |  | % within Disease Course | 40.7% | 39.9% | 19.4% | 100% |
|  |  | Secondary progressive | No. of Cases | 123 | 161 | 75 | 359 |
|  |  |  | % within Disease Course | 34.3% | 44.8% | 20.9% | 100% |
|  |  | Don't know | No. of Cases | 272 | 261 | 125 | 658 |
|  |  |  | % within Disease Course | 41.3% | 39.7% | 19.0% | 100% |
|  |  | Total | No. of Cases | 1771 | 1778 | 855 | 4404 |
|  |  |  | % within Disease Course | 40.2% | 40.4% | 19.4% | 100% |
