# Supplementary material for: Physical Disability, Anxiety and Depression in People with MS: An Internet-Based Survey via the UK MS Register
Source: PLoS One. 2014 Aug 25;9(8):e104604. doi: 10.1371/journal.pone.0104604 (PMC4143231; doi:10.1371/journal.pone.0104604)
Supplement: Table S4 — Proportions and frequencies: HADS-D. This shows the proportions and frequencies of respondents reporting normal (scores up to 7), mild (8–10), moderate (11–15) and severe depression (over 15), stratified by age, gender and disease course. (DOCX) [file pone.0104604.s004.docx]

**Supplementary Table D.**

| Age Band | Gender | Disease Course | | Depression | | | | Total |
| --- | --- | --- | --- | --- | --- | --- | --- | --- |
|  |  |  |  | Normal | Mild | Moderate | Severe |  |
| <= 39 | Male | Primary progressive | No. of Cases | 3 | 5 | 3 | 0 | 11 |
|  |  |  | % within Disease Course | 27.3% | 45.5% | 27.3% | 0.0% | 100% |
|  |  | Relapsing-remitting | No. of Cases | 86 | 28 | 16 | 1 | 131 |
|  |  |  | % within Disease Course | 65.6% | 21.4% | 12.2% | 0.8% | 100% |
|  |  | Secondary progressive | No. of Cases | 1 | 1 | 1 | 0 | 3 |
|  |  |  | % within Disease Course | 33.3% | 33.3% | 33.3% | 0.0% | 100% |
|  |  | Don't know | No. of Cases | 7 | 7 | 3 | 0 | 17 |
|  |  |  | % within Disease Course | 41.2% | 41.2% | 17.6% | 0.0% | 100% |
|  |  | Total | No. of Cases | 97 | 41 | 23 | 1 | 162 |
|  |  |  | % within Disease Course | 59.9% | 25.3% | 14.2% | 0.6% | 100% |
|  | Female | Primary progressive | No. of Cases | 8 | 5 | 3 | 1 | 17 |
|  |  |  | % within Disease Course | 47.1% | 29.4% | 17.6% | 5.9% | 100% |
|  |  | Relapsing-remitting | No. of Cases | 336 | 122 | 61 | 12 | 531 |
|  |  |  | % within Disease Course | 63.3% | 23.0% | 11.5% | 2.3% | 100% |
|  |  | Secondary progressive | No. of Cases | 3 | 1 | 0 | 0 | 4 |
|  |  |  | % within Disease Course | 75.0% | 25.0% | 0.0% | 0.0% | 100% |
|  |  | Don't know | No. of Cases | 21 | 8 | 5 | 5 | 39 |
|  |  |  | % within Disease Course | 53.8% | 20.5% | 12.8% | 12.8% | 100% |
|  |  | Total | No. of Cases | 368 | 136 | 69 | 18 | 591 |
|  |  |  | % within Disease Course | 62.3% | 23.0% | 11.7% | 3.0% | 100% |
|  | Total | Primary progressive | No. of Cases | 11 | 10 | 6 | 1 | 28 |
|  |  |  | % within Disease Course | 39.3% | 35.7% | 21.4% | 3.6% | 100% |
|  |  | Relapsing-remitting | No. of Cases | 422 | 150 | 77 | 13 | 662 |
|  |  |  | % within Disease Course | 63.7% | 22.7% | 11.6% | 2.0% | 100% |
|  |  | Secondary progressive | No. of Cases | 4 | 2 | 1 | 0 | 7 |
|  |  |  | % within Disease Course | 57.1% | 28.6% | 14.3% | 0.0% | 100% |
|  |  | Don't know | No. of Cases | 28 | 15 | 8 | 5 | 56 |
|  |  |  | % within Disease Course | 50.0% | 26.8% | 14.3% | 8.9% | 100% |
|  |  | Total | No. of Cases | 465 | 177 | 92 | 19 | 753 |
|  |  |  | % within Disease Course | 61.8% | 23.5% | 12.2% | 2.5% | 100% |
| 40 to 49 | Male | Primary progressive | No. of Cases | 24 | 13 | 14 | 4 | 55 |
|  |  |  | % within Disease Course | 43.6% | 23.6% | 25.5% | 7.3% | 100% |
|  |  | Relapsing-remitting | No. of Cases | 115 | 76 | 27 | 10 | 228 |
|  |  |  | % within Disease Course | 50.4% | 33.3% | 11.8% | 4.4% | 100% |
|  |  | Secondary progressive | No. of Cases | 3 | 7 | 10 | 3 | 23 |
|  |  |  | % within Disease Course | 13.0% | 30.4% | 43.5% | 13.0% | 100% |
|  |  | Don't know | No. of Cases | 4 | 13 | 5 | 6 | 28 |
|  |  |  | % within Disease Course | 14.3% | 46.4% | 17.9% | 21.4% | 100% |
|  |  | Total | No. of Cases | 146 | 109 | 56 | 23 | 334 |
|  |  |  | % within Disease Course | 43.7% | 32.6% | 16.8% | 6.9% | 100% |
|  | Female | Primary progressive | No. of Cases | 24 | 21 | 14 | 2 | 61 |
|  |  |  | % within Disease Course | 39.3% | 34.4% | 23.0% | 3.3% | 100% |
|  |  | Relapsing-remitting | No. of Cases | 415 | 199 | 96 | 23 | 733 |
|  |  |  | % within Disease Course | 56.6% | 27.1% | 13.1% | 3.1% | 100% |
|  |  | Secondary progressive | No. of Cases | 12 | 13 | 11 | 3 | 39 |
|  |  |  | % within Disease Course | 30.8% | 33.3% | 28.2% | 7.7% | 100% |
|  |  | Don't know | No. of Cases | 53 | 35 | 13 | 7 | 108 |
|  |  |  | % within Disease Course | 49.1% | 32.4% | 12.0% | 6.5% | 100% |
|  |  | Total | No. of Cases | 504 | 268 | 134 | 35 | 941 |
|  |  |  | % within Disease Course | 53.6% | 28.5% | 14.2% | 3.7% | 100% |
|  | Total | Primary progressive | No. of Cases | 48 | 34 | 28 | 6 | 116 |
|  |  |  | % within Disease Course | 41.4% | 29.3% | 24.1% | 5.2% | 100% |
|  |  | Relapsing-remitting | No. of Cases | 530 | 275 | 123 | 33 | 961 |
|  |  |  | % within Disease Course | 55.2% | 28.6% | 12.8% | 3.4% | 100% |
|  |  | Secondary progressive | No. of Cases | 15 | 20 | 21 | 6 | 62 |
|  |  |  | % within Disease Course | 24.2% | 32.3% | 33.9% | 9.7% | 100% |
|  |  | Don't know | No. of Cases | 57 | 48 | 18 | 13 | 136 |
|  |  |  | % within Disease Course | 41.9% | 35.3% | 13.2% | 9.6% | 100% |
|  |  | Total | No. of Cases | 650 | 377 | 190 | 58 | 1275 |
|  |  |  | % within Disease Course | 51.0% | 29.6% | 14.9% | 4.5% | 100% |
| 50 to 59 | Male | Primary progressive | No. of Cases | 41 | 25 | 21 | 7 | 94 |
|  |  |  | % within Disease Course | 43.6% | 26.6% | 22.3% | 7.4% | 100% |
|  |  | Relapsing-remitting | No. of Cases | 89 | 46 | 36 | 9 | 180 |
|  |  |  | % within Disease Course | 49.4% | 25.6% | 20.0% | 5.0% | 100% |
|  |  | Secondary progressive | No. of Cases | 15 | 22 | 14 | 4 | 55 |
|  |  |  | % within Disease Course | 27.3% | 40.0% | 25.5% | 7.3% | 100% |
|  |  | Don't know | No. of Cases | 28 | 14 | 14 | 3 | 59 |
|  |  |  | % within Disease Course | 47.5% | 23.7% | 23.7% | 5.1% | 100% |
|  |  | Total | No. of Cases | 173 | 107 | 85 | 23 | 388 |
|  |  |  | % within Disease Course | 44.6% | 27.6% | 21.9% | 5.9% | 100% |
|  | Female | Primary progressive | No. of Cases | 60 | 38 | 22 | 9 | 129 |
|  |  |  | % within Disease Course | 46.5% | 29.5% | 17.1% | 7.0% | 100% |
|  |  | Relapsing-remitting | No. of Cases | 323 | 179 | 75 | 19 | 596 |
|  |  |  | % within Disease Course | 54.2% | 30.0% | 12.6% | 3.2% | 100% |
|  |  | Secondary progressive | No. of Cases | 30 | 25 | 17 | 3 | 75 |
|  |  |  | % within Disease Course | 40.0% | 33.3% | 22.7% | 4.0% | 100% |
|  |  | Don't know | No. of Cases | 83 | 37 | 36 | 6 | 162 |
|  |  |  | % within Disease Course | 51.2% | 22.8% | 22.2% | 3.7% | 100% |
|  |  | Total | No. of Cases | 496 | 279 | 150 | 37 | 962 |
|  |  |  | % within Disease Course | 51.6% | 29.0% | 15.6% | 3.8% | 100% |
|  | Total | Primary progressive | No. of Cases | 101 | 63 | 43 | 16 | 223 |
|  |  |  | % within Disease Course | 45.3% | 28.3% | 19.3% | 7.2% | 100% |
|  |  | Relapsing-remitting | No. of Cases | 412 | 225 | 111 | 28 | 776 |
|  |  |  | % within Disease Course | 53.1% | 29.0% | 14.3% | 3.6% | 100% |
|  |  | Secondary progressive | No. of Cases | 45 | 47 | 31 | 7 | 130 |
|  |  |  | % within Disease Course | 34.6% | 36.2% | 23.8% | 5.4% | 100% |
|  |  | Don't know | No. of Cases | 111 | 51 | 50 | 9 | 221 |
|  |  |  | % within Disease Course | 50.2% | 23.1% | 22.6% | 4.1% | 100% |
|  |  | Total | No. of Cases | 669 | 386 | 235 | 60 | 1350 |
|  |  |  | % within Disease Course | 49.6% | 28.6% | 17.4% | 4.4% | 100% |
| 60 to 69 | Male | Primary progressive | No. of Cases | 65 | 32 | 18 | 6 | 121 |
|  |  |  | % within Disease Course | 53.7% | 26.4% | 14.9% | 5.0% | 100% |
|  |  | Relapsing-remitting | No. of Cases | 60 | 20 | 14 | 5 | 99 |
|  |  |  | % within Disease Course | 60.6% | 20.2% | 14.1% | 5.1% | 100% |
|  |  | Secondary progressive | No. of Cases | 24 | 16 | 7 | 2 | 49 |
|  |  |  | % within Disease Course | 49.0% | 32.7% | 14.3% | 4.1% | 100% |
|  |  | Don't know | No. of Cases | 28 | 20 | 5 | 2 | 55 |
|  |  |  | % within Disease Course | 50.9% | 36.4% | 9.1% | 3.6% | 100% |
|  |  | Total | No. of Cases | 177 | 88 | 44 | 15 | 324 |
|  |  |  | % within Disease Course | 54.6% | 27.2% | 13.6% | 4.6% | 100% |
|  | Female | Primary progressive | No. of Cases | 72 | 19 | 11 | 1 | 103 |
|  |  |  | % within Disease Course | 69.9% | 18.4% | 10.7% | 1.0% | 100% |
|  |  | Relapsing-remitting | No. of Cases | 128 | 63 | 17 | 2 | 210 |
|  |  |  | % within Disease Course | 61.0% | 30.0% | 8.1% | 1.0% | 100% |
|  |  | Secondary progressive | No. of Cases | 47 | 22 | 8 | 2 | 79 |
|  |  |  | % within Disease Course | 59.5% | 27.8% | 10.1% | 2.5% | 100% |
|  |  | Don't know | No. of Cases | 79 | 36 | 19 | 4 | 138 |
|  |  |  | % within Disease Course | 57.2% | 26.1% | 13.8% | 2.9% | 100% |
|  |  | Total | No. of Cases | 326 | 140 | 55 | 9 | 530 |
|  |  |  | % within Disease Course | 61.5% | 26.4% | 10.4% | 1.7% | 100% |
|  | Total | Primary progressive | No. of Cases | 137 | 51 | 29 | 7 | 224 |
|  |  |  | % within Disease Course | 61.2% | 22.8% | 12.9% | 3.1% | 100% |
|  |  | Relapsing-remitting | No. of Cases | 188 | 83 | 31 | 7 | 309 |
|  |  |  | % within Disease Course | 60.8% | 26.9% | 10.0% | 2.3% | 100% |
|  |  | Secondary progressive | No. of Cases | 71 | 38 | 15 | 4 | 128 |
|  |  |  | % within Disease Course | 55.5% | 29.7% | 11.7% | 3.1% | 100% |
|  |  | Don't know | No. of Cases | 107 | 56 | 24 | 6 | 193 |
|  |  |  | % within Disease Course | 55.4% | 29.0% | 12.4% | 3.1% | 100% |
|  |  | Total | No. of Cases | 503 | 228 | 99 | 24 | 854 |
|  |  |  | % within Disease Course | 58.9% | 26.7% | 11.6% | 2.8% | 100% |
| >=70 | Male | Primary progressive | No. of Cases | 18 | 5 | 3 | 1 | 27 |
|  |  |  | % within Disease Course | 66.7% | 18.5% | 11.1% | 3.7% | 100% |
|  |  | Relapsing-remitting | No. of Cases | 5 | 3 | 2 | 0 | 10 |
|  |  |  | % within Disease Course | 50.0% | 30.0% | 20.0% | 0.0% | 100% |
|  |  | Secondary progressive | No. of Cases | 4 | 2 | 3 | 0 | 9 |
|  |  |  | % within Disease Course | 44.4% | 22.2% | 33.3% | 0.0% | 100% |
|  |  | Don't know | No. of Cases | 12 | 7 | 2 | 0 | 21 |
|  |  |  | % within Disease Course | 57.1% | 33.3% | 9.5% | 0.0% | 100% |
|  |  | Total | No. of Cases | 39 | 17 | 10 | 1 | 67 |
|  |  |  | % within Disease Course | 58.2% | 25.4% | 14.9% | 1.5% | 100% |
|  | Female | Primary progressive | No. of Cases | 22 | 9 | 1 | 0 | 32 |
|  |  |  | % within Disease Course | 68.8% | 28.1% | 3.1% | 0.0% | 100% |
|  |  | Relapsing-remitting | No. of Cases | 11 | 6 | 1 | 1 | 19 |
|  |  |  | % within Disease Course | 57.9% | 31.6% | 5.3% | 5.3% | 100% |
|  |  | Secondary progressive | No. of Cases | 12 | 7 | 4 | 0 | 23 |
|  |  |  | % within Disease Course | 52.2% | 30.4% | 17.4% | 0.0% | 100% |
|  |  | Don't know | No. of Cases | 26 | 3 | 2 | 0 | 31 |
|  |  |  | % within Disease Course | 83.9% | 9.7% | 6.5% | 0.0% | 100% |
|  |  | Total | No. of Cases | 71 | 25 | 8 | 1 | 105 |
|  |  |  | % within Disease Course | 67.6% | 23.8% | 7.6% | 1.0% | 100% |
|  | Total | Primary progressive | No. of Cases | 40 | 14 | 4 | 1 | 59 |
|  |  |  | % within Disease Course | 67.8% | 23.7% | 6.8% | 1.7% | 100% |
|  |  | Relapsing-remitting | No. of Cases | 16 | 9 | 3 | 1 | 29 |
|  |  |  | % within Disease Course | 55.2% | 31.0% | 10.3% | 3.4% | 100% |
|  |  | Secondary progressive | No. of Cases | 16 | 9 | 7 | 0 | 32 |
|  |  |  | % within Disease Course | 50.0% | 28.1% | 21.9% | 0.0% | 100% |
|  |  | Don't know | No. of Cases | 38 | 10 | 4 | 0 | 52 |
|  |  |  | % within Disease Course | 73.1% | 19.2% | 7.7% | 0.0% | 100% |
|  |  | Total | No. of Cases | 110 | 42 | 18 | 2 | 172 |
|  |  |  | % within Disease Course | 64.0% | 24.4% | 10.5% | 1.2% | 100% |
| All Ages | Male | Primary progressive | No. of Cases | 151 | 80 | 59 | 18 | 308 |
|  |  |  | % within Disease Course | 49.0% | 26.0% | 19.2% | 5.8% | 100% |
|  |  | Relapsing-remitting | No. of Cases | 355 | 173 | 95 | 25 | 648 |
|  |  |  | % within Disease Course | 54.8% | 26.7% | 14.7% | 3.9% | 100% |
|  |  | Secondary progressive | No. of Cases | 47 | 48 | 35 | 9 | 139 |
|  |  |  | % within Disease Course | 33.8% | 34.5% | 25.2% | 6.5% | 100% |
|  |  | Don't know | No. of Cases | 79 | 61 | 29 | 11 | 180 |
|  |  |  | % within Disease Course | 43.9% | 33.9% | 16.1% | 6.1% | 100% |
|  |  | Total | No. of Cases | 632 | 362 | 218 | 63 | 1275 |
|  |  |  | % within Disease Course | 49.6% | 28.4% | 17.1% | 4.9% | 100% |
|  | Female | Primary progressive | No. of Cases | 186 | 92 | 51 | 13 | 342 |
|  |  |  | % within Disease Course | 54.4% | 26.9% | 14.9% | 3.8% | 100% |
|  |  | Relapsing-remitting | No. of Cases | 1213 | 569 | 250 | 57 | 2089 |
|  |  |  | % within Disease Course | 58.1% | 27.2% | 12.0% | 2.7% | 100% |
|  |  | Secondary progressive | No. of Cases | 104 | 68 | 40 | 8 | 220 |
|  |  |  | % within Disease Course | 47.3% | 30.9% | 18.2% | 3.6% | 100% |
|  |  | Don't know | No. of Cases | 262 | 119 | 75 | 22 | 478 |
|  |  |  | % within Disease Course | 54.8% | 24.9% | 15.7% | 4.6% | 100% |
|  |  | Total | No. of Cases | 1765 | 848 | 416 | 100 | 3129 |
|  |  |  | % within Disease Course | 56.4% | 27.1% | 13.3% | 3.2% | 100% |
|  | Total | Primary progressive | No. of Cases | 337 | 172 | 110 | 31 | 650 |
|  |  |  | % within Disease Course | 51.8% | 26.5% | 16.9% | 4.8% | 100% |
|  |  | Relapsing-remitting | No. of Cases | 1568 | 742 | 345 | 82 | 2737 |
|  |  |  | % within Disease Course | 57.3% | 27.1% | 12.6% | 3.0% | 100% |
|  |  | Secondary progressive | No. of Cases | 151 | 116 | 75 | 17 | 359 |
|  |  |  | % within Disease Course | 42.1% | 32.3% | 20.9% | 4.7% | 100% |
|  |  | Don't know | No. of Cases | 341 | 180 | 104 | 33 | 658 |
|  |  |  | % within Disease Course | 51.8% | 27.4% | 15.8% | 5.0% | 100% |
|  |  | Total | No. of Cases | 2397 | 1210 | 634 | 163 | 4404 |
|  |  |  | % within Disease Course | 54.4% | 27.5% | 14.4% | 3.7% | 100% |
